# Trichobezoar as an Unusual Cause for Iron Deficiency Anemia: A Rare Case Report

**DOI:** 10.1002/ccr3.70186

**Published:** 2025-02-10

**Authors:** Mohamad Moamen Almouallem, Majd Hanna, Nafiza Martini, Ahmad Alfarouh, Rand Mousseli, Maysam Yaldany, Jaber Mahmod

**Affiliations:** ^1^ Faculty of Medicine Damascus University Damascus Syrian Arab Republic; ^2^ Stemosis for Scientific Research Damascus Syrian Arab Republic

**Keywords:** abdominal pain, anemia, bezoars, pediatrics, surgery, vomiting

## Abstract

Trichobezoars are conglomerations of undigested foreign materials within the gastrointestinal tract (GIT). They are most commonly observed in young girls. Trichobezoars typically lodge in the stomach, but can extend further into the intestines in a condition called Rapunzel syndrome. The current patient, a young girl, complained of abdominal pain accompanied by diarrhea. She also had a history of bile vomiting 1 month prior and an abnormal craving for mud (PICA). Trichobezoars are often linked to hair‐pulling disorders or other mental health conditions, and symptoms can be vague for years before progressing to nausea, vomiting, weight loss, and even iron deficiency anemia. Treatment involves removing the hairball and addressing any underlying psychological issues. In the current case, we aimed to elucidate the diagnosis, management, and unique features of gastrointestinal tract trichobezoars, which are uncommon causes of gastrointestinal distress and iron deficiency anemia (IDA).

**Summary**

- Bezoars are rare collections of hard non‐digestible foreign bodies that usually accumulate in the stomach and/or other areas of the gastrointestinal tract.
- They can cause iron deficiency anemia, especially in young females with a history of hair ingestion.
- Thus, early suspicion and diagnosis are crucial for preventing complication.

## 1 Introduction

Bezoars, such as trichobezoars, are clusters of undigested foreign substances found in the gastrointestinal tract (GIT). Trichobezoars in the GIT are exceptionally uncommon, representing approximately 6% of all bezoars. Factors that increase the risk of trichobezoars include mental health conditions such as trichotillomania (hair pulling), trichophagia (hair consumption), depression, anxiety, and poor self‐esteem [1, 2].

Trichobezoars typically reside in the stomach and can remain symptomless for years until complications arise due to the stomach's high capacity. In some instances, they can extend into the jejunum, ileum, and colon, forming a tail‐like structure known as Rapunzel syndrome, which is an incredibly uncommon side effect of a gastric trichobezoar. Thus, symptoms can vary, including abdominal pain and vomiting [1, 3, 4, 5, 6]. Complications may involve obstruction and gastrointestinal perforation [1]. However, iron deficiency anemia is considered a complication of trichobezoar [2].

The current report describes the case of an 11‐year‐old girl who was diagnosed with a GIT trichobezoar leading to iron deficiency anemia (IDA). By sharing the current case, we aim to raise awareness among healthcare professionals and stimulate discussions on uncommon causes of anemia and abdominal symptoms.

## 2 Case History/Examination

An 11‐year‐old girl presented by her parents to the Children's Hospital with a history of epigastric pain, accompanied by frequent diarrhea and bile vomiting, which started a month ago with unknown details. A gastroenterologist in an outpatient clinic ordered blood tests, including Complete Blood Count, Ferritin, and C‐reactive protein. The results showed a drop in hemoglobin levels to 8 g/dL (Reference range: 11.9–15.0 g/dL), a decrease in ferritin values to 3.97 ng/mL (Reference range: 7–140 ng/mL), and an increase in CRP levels to 0.82 mg/dL (normal/minor elevation: 0.3–1.0 mg/dL). Based on these findings, the doctor diagnosed the patient with Iron deficiency anemia and prescribed oral iron supplements, which resulted in her gradual recovery.

However, the same symptoms reoccurred 10 days later, along with the presence of clay‐colored stools. This change in stool color may be a side effect of the iron supplements. However, due to her relapse and recurrent symptoms, she was referred to the Children's Hospital. Upon clinical examination, her consciousness was normal, but she exhibited abdominal tenderness. She appeared pale with white nails, despite having a good appetite. Additionally, during the investigation, the parents disclosed that the girl had a habit of hair ingestion (Trichphagia) since she was 7 years old. However, psychiatric illness was not confirmed. The girl was referred to the psychiatry department, but regrettably, her parents did not follow through with the referral. This may have been due to their limited understanding or awareness of psychological disorders in children.

## 3 Differential Diagnosis, Investigation, and Treatment

**FIGURE 1:**
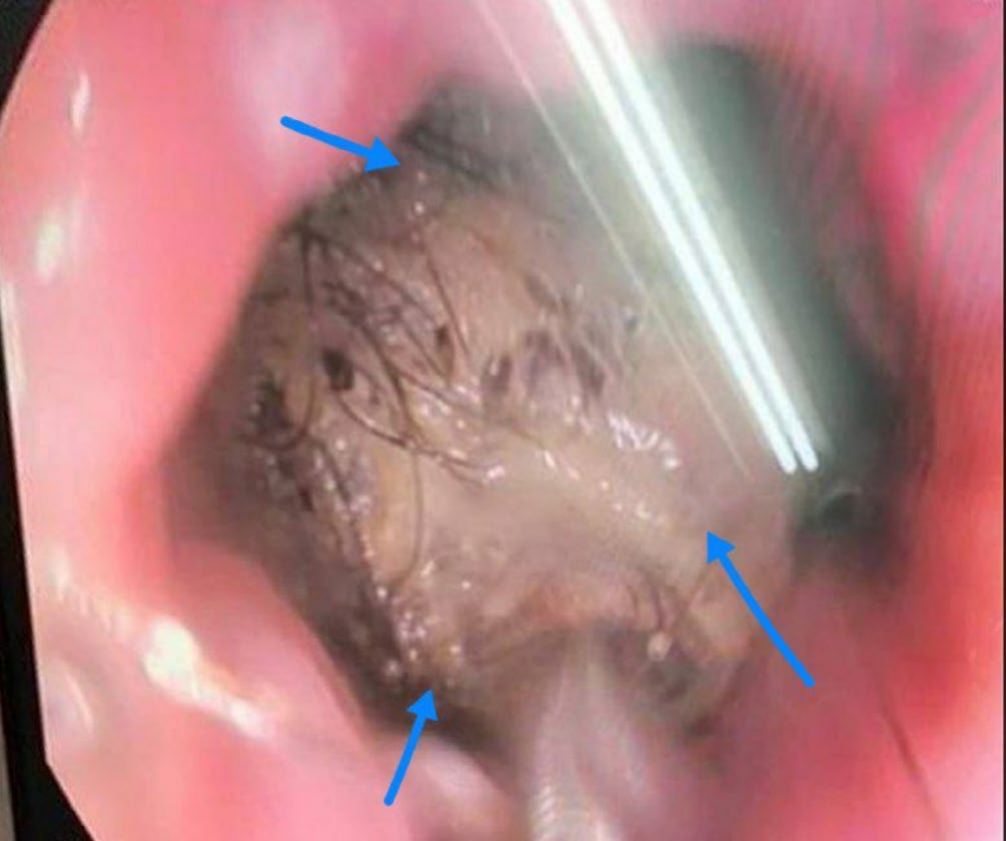
The mass of hair observed during upper gastrointestinal endoscopy.

**FIGURE 2:**
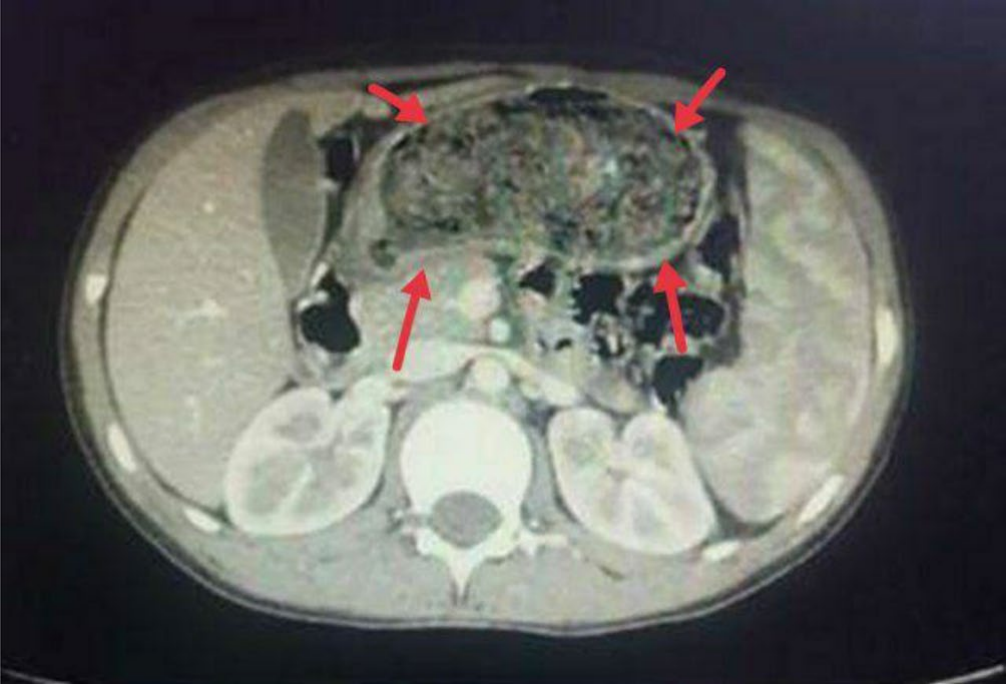
CT scan of the abdomen showing the mass of hair filling the entire stomach.

**FIGURE 3:**
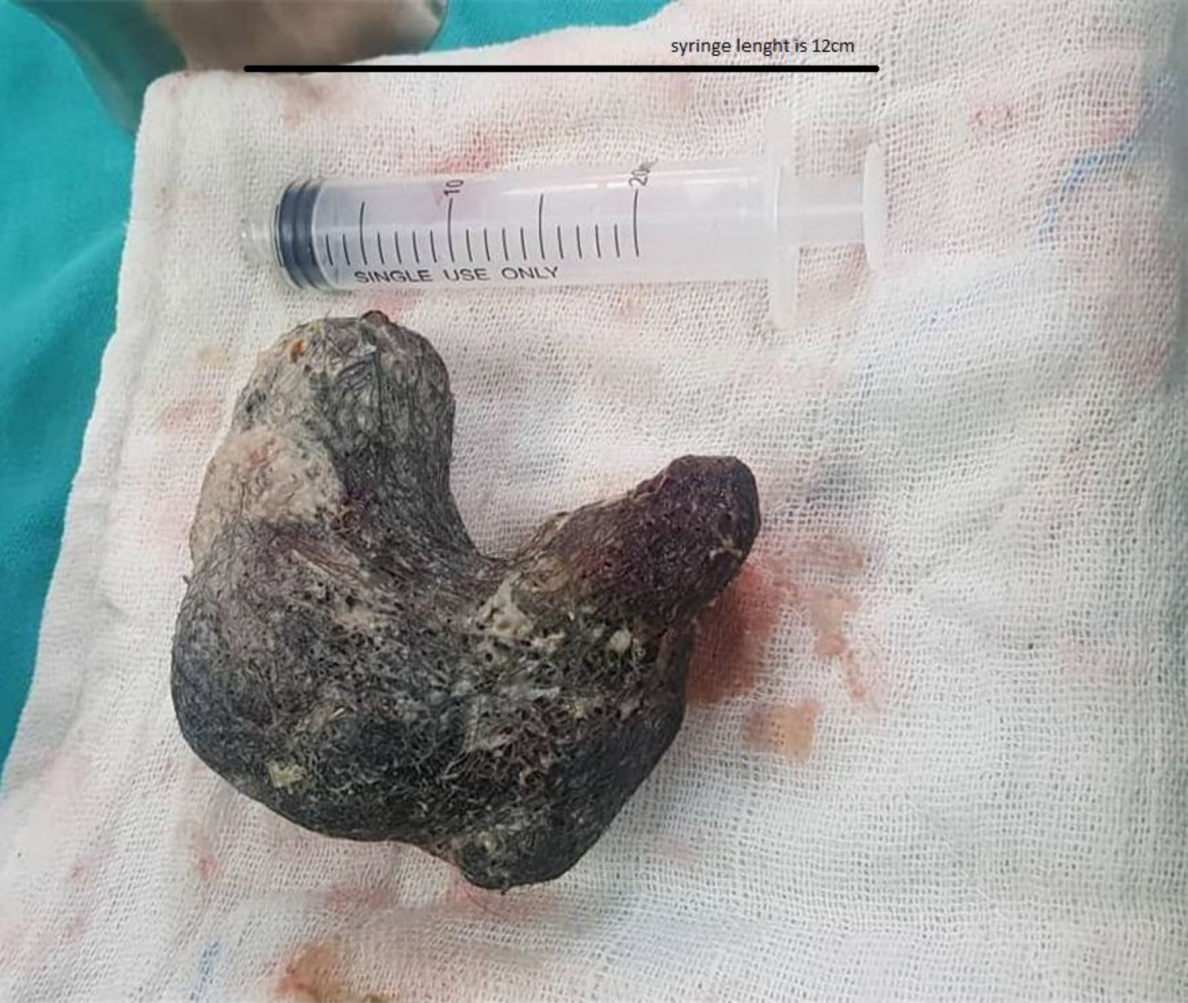
The large, hard mass of hair and ingested foreign bodies extracted from the stomach after surgery, with its size compared to that of a syringe.

Ultrasound examination revealed a hypoechoic mass occupying two‐thirds of the stomach and extending into the duodenum. An Upper gastrointestinal endoscopy was performed, which revealed a mass of hair filling the entire stomach. Subsequently, a CT scan was conducted to assess the extent of the hair mass (Figures 1 and 2). Despite attempts to remove the hair mass through endoscopy, the procedure was unsuccessful. Consequently, the choice was made to remove the hair mass, which was blocking the exit of the stomach, by laparotomy (Figure 3).

Following the surgical operation, the patient was placed on observation for 7 days, during which she was kept on a nothing‐by‐mouth (NPO) status and received nutrition through a nasogastric tube (NGT). She was also administered painkillers and antibiotics (linezolid and ciprofloxacin).

## 4 Outcome and Follow‐Up

No complications occurred after the surgical operation. The girl was discharged 22 days after admission, with a medical prescription for iron supplements at a dosage of 3 mg per kg. After being discharged from the hospital, the girl was reassessed 2 weeks later and found to be in good health. Her hemoglobin levels and ferritin values had returned to their normal, healthy ranges during this time. Since that follow‐up visit, she has not returned to the hospital.

## 5 Discussion

Bezoars are rare clumps of undigestible material that can form in the digestive system [2, 7, 8]. Based on their major constituents, bezoars are categorized into multiple categories. The most prevalent ones are lactobezoar (milk formula), trichobezoar (hair), and phytobezoar (vegetable).

Trichobezoars are hair‐based and most common in young females with hair‐related disorders. The current case is of a young girl with a trichobezoar in her stomach that extended to the duodenum [2].

Trichobezoars typically occur in young girls with psychiatric conditions like trichophagia (hair ingestion) and trichotillomania (compulsive hair pulling). Trichotillomania affects approximately 1 in 2000 children globally, with approximately 30% of these children also experiencing trichophagia. Among those suffering from trichophagia, only 1% will develop a trichobezoar [2]. However, psychiatric illness was not confirmed in the current case. GIT trichobezoars represent less than 6% of bezoars, and up to 90% of cases have been documented in females between the ages of 13 and 20 [9]. Similarly, the current case was young (11 years old) and female.

Normally found in the stomach, trichobezoars can also extend to the small intestine or even the colon in approximately 10% of cases. This disease is known as Rapunzel syndrome [2, 10]. In the current case, the mass occupied two‐thirds of the stomach and extended to the duodenum as confirmed by tomography. However, most opinions [11, 12, 13, 14, 15, 16] have indicated that in patients with Rapunzel syndrome, the tail has at least reached the jejunum.

The size and location of GI trichobezoars determine their clinical presentation. Years may pass before a diagnosis is made despite vague symptoms such as nausea, vomiting, and stomach discomfort [9]. Enlargement of a trichobezoar can cause symptoms like epigastric discomfort, abdominal pain, nausea, vomiting, weight loss, fatigue, constipation (more common), and diarrhea [2, 9, 17, 18].

Trichobezoars are predisposed to mental illnesses such as trichotillomania, trichophagia, anxiety, sadness, and low self‐esteem [2]. The current case had habits of hair pulling and eating as well.

Additional symptoms may include halitosis, peritonitis, anorexia, and pain associated with eating. Due to the difficulties of trichobezoars, there may also be signs of malabsorption and iron deficiency anemia (IDA) [2, 6, 19].

Trichobezoars do not commonly cause IDA for the following reasons:

- Iron absorption location: the primary site for iron absorption is the duodenum and proximal jejunum. Trichobezoars typically reside in the stomach and may not significantly interfere with iron absorption in these lower segments of the GIT [6].
- Gastric mucosa integrity: unlike other causes of IDA (such as chronic gastrointestinal bleeding), trichobezoars do not necessarily damage the gastric mucosa. Therefore, they do not directly lead to significant blood loss or iron deficiency [20].

The current case presented to the emergency department with abdominal pain, iron deficiency anemia, and a history of vomiting. In the reported case, the trichobezoar was an unusual cause of anemia and abdominal pain, which underscores the importance of bezoars as a differential diagnosis for such cases of vague symptoms.

A trichobezoar may not cause any symptoms, which could cause a delayed presentation. If untreated for an extended period of time, it can cause severe anemia due to either malabsorption or gastrointestinal bleeding. Failure to thrive may arise from this. Additionally, since imaging is the foundation of diagnosis, radiologists and physicians must be extremely cautious in order to identify the illness early and avoid major consequences [2]. The current case presented late with these symptoms. Ultrasound is often unhelpful, but CT scans show a well‐defined mass [6]. Treatment involves addressing the hair‐eating habit and removing the bezoar, usually through surgery (laparotomy) [2]. The current case involved a large mass requiring surgery.

Psychiatric counseling has a significant role in preventing bezoar recurrence because many of these individuals suffer from mental problems such as eating disorders, emotional difficulties, and mental retardation [6].

Despite the lack of studies on the use of medication for treating trichotillomania, some people seem to respond to fluoxetine or other serotonin reuptake inhibitors. One more element of treatment targeted at reducing recurrence is routine parent counseling. Patients are said to have a good long‐term prognosis when behavioral therapy is used to reduce trichophagia and psychological/psychiatric follow‐up is maintained [6].

## 6 Conclusion

The current case raises the awareness of trichobezoars as an unusual diagnosis for iron deficiency anemia (IDA) in young females who present with diarrhea, IDA, clay‐colored stools, and a history of vomiting. Additionally, we underscore the importance of psychiatric counseling in preventing bezoar recurrence.

## Author Contributions

**Mohamad Moamen Almouallem:** conceptualization, writing – original draft, writing – review and editing. **Majd Hanna:** data curation, investigation, writing – original draft. **Nafiza Martini:** writing – original draft. **Ahmad Alfarouh:** writing – original draft. **Rand Mousseli:** writing – original draft. **Maysam Yaldany:** writing – original draft. **Jaber Mahmod:** conceptualization, resources, supervision, writing – review and editing.

## Consent

In accordance with the journal's patient consent policy, the patient provided written informed consent for the publication of this report.

## Conflicts of Interest

The authors declare no conflicts of interest.

## Data Availability

The data that support the findings of this study are available on request from the corresponding author.
